# EMTome: a resource for pan-cancer analysis of epithelial-mesenchymal transition genes and signatures

**DOI:** 10.1038/s41416-020-01178-9

**Published:** 2020-12-10

**Authors:** Suhas V. Vasaikar, Abhijeet P. Deshmukh, Petra den Hollander, Sridevi Addanki, Nick Allen Kuburich, Sriya Kudaravalli, Robiya Joseph, Jeffrey T. Chang, Rama Soundararajan, Sendurai A. Mani

**Affiliations:** 1 Department of Translational Molecular Pathology, Division of Pathology/Lab Medicine, The University of Texas MD Anderson Cancer Center, Houston, TX 77030 USA; 2 Department of Integrative Biology & Pharmacology, Institute of Molecular Medicine, School of Biomedical Informatics University of Texas Health Science Center at Houston, Houston, TX 77030 USA

**Keywords:** Metastasis, Tumour-suppressor proteins

## Abstract

**Background:**

The epithelial-mesenchymal transition (EMT) enables dissociation of tumour cells from the primary tumour mass, invasion through the extracellular matrix, intravasation into blood vessels and colonisation of distant organs. Cells that revert to the epithelial state via the mesenchymal-epithelial transition cause metastases, the primary cause of death in cancer patients. EMT also empowers cancer cells with stem-cell properties and induces resistance to chemotherapeutic drugs. Understanding the driving factors of EMT is critical for the development of effective therapeutic interventions.

**Methods:**

This manuscript describes the generation of a database containing EMT gene signatures derived from cell lines, patient-derived xenografts and patient studies across cancer types and multiomics data and the creation of a web-based portal to provide a comprehensive analysis resource.

**Results:**

EMTome incorporates (i) EMT gene signatures; (ii) EMT-related genes with multiomics features across different cancer types; (iii) interactomes of EMT-related genes (miRNAs, transcription factors, and proteins); (iv) immune profiles identified from The Cancer Genome Atlas (TCGA) cohorts by exploring transcriptomics, epigenomics, and proteomics, and drug sensitivity and (iv) clinical outcomes of cancer cohorts linked to EMT gene signatures.

**Conclusion:**

The web-based EMTome portal is a resource for primary and metastatic tumour research publicly available at www.emtome.org.

## Background

Transdifferentiation between epithelial and mesenchymal states is a fundamental cellular process necessary during embryonic development.^1,2^ During epithelial-mesenchymal transition (EMT), epithelial cells lose tight cell–cell connections and polarity, which confers migratory and invasive properties. In the reverse process referred to as mesenchymal-epithelial transition (MET), cells loose migratory freedom, begin expressing junction complexes and adopt apicobasal polarity.^3^ EMT and MET are critical for embryonic development, tissue regeneration and wound healing but also contribute to organ fibrosis, cancer progression and metastasis.^4,5^

**Fig. 1:**
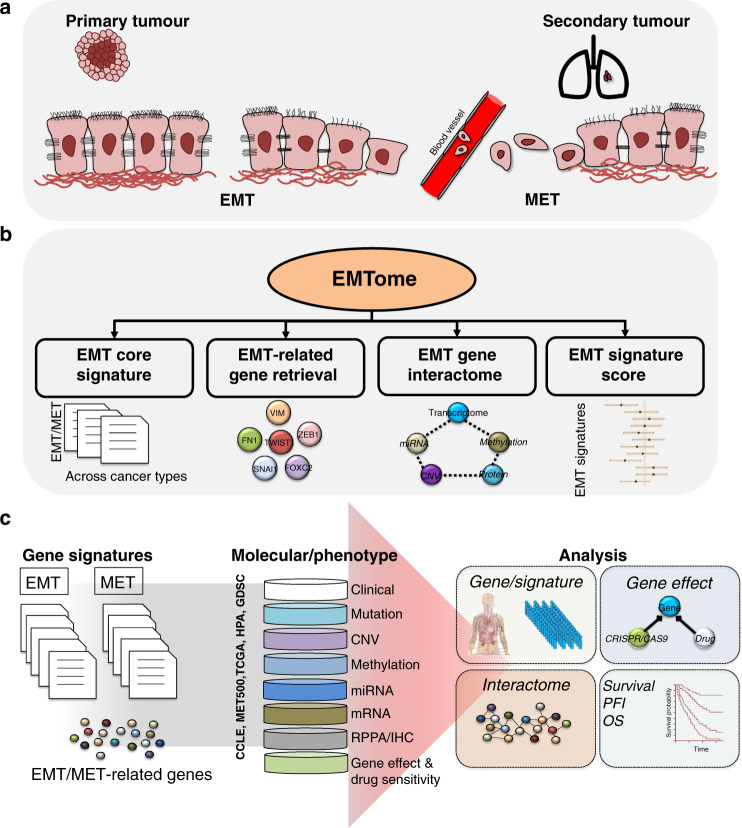
Schematic overview of the EMTome. **a** The transformation between epithelial and mesenchymal cells during cancer metastasis is orchestrated through multiple genomic changes that eventually lead to primary site escape, intravasation and formation of a tumour at a secondary site. **b** Schematic of the EMTome database. The EMT core signature module allows the user to explore EMT signatures and the markers associated with each signature across cancer types. The EMT-related gene retrieval module can be used to explore EMT-related genes for changes at the transcriptomic, epigenomic or proteomic level. The EMT-related gene interactome module can be used to search for significant associations between gene RNA expression and the transcriptomic landscape, copy number events, methylation, miRNA and proteins (RPPA). The fourth module is the EMT-signature score module. **c** The EMT-signature score module facilitates calculation of correlations between the normalised EMT score and RNA expression, survival comparisons across cancer types, and evaluation of genetic dependency of EMT-related genes and available drug sensitivity information.

It is now widely accepted that EMT and MET are activated during cancer progression. The EMT confers properties that result in dissociation of tumour cells from the primary tumour mass, invasion through the extracellular matrix, intravasation into blood vessels and colonisation of distant organs, and re-activation of epithelial properties at the secondary site via the MET cause metastases, the primary cause of death in cancer patients (Fig. 1a). Identifying the EMT state in a tumour is a challenging process due to the transient and reversible nature of the process. The expression of E-cadherin, Epcam, claudins, occludins and cytokeratins are commonly used markers of the epithelial state, whereas expression of vimentin (VIM), fibronectin and α-SMA are markers of the mesenchymal state.^6^ EpCAM-based enrichment techniques are commonly used to detect epithelial tumour progression; however, these markers fail to enrich for tumour cells that have undergone EMT such as circulating tumour cells of prostate cancer, ovarian and breast cancer.^7^

In 1994, Miettinen et al. showed that the treatment of cuboidal epithelial cells with TGF-β reversibly induced differentiation into a fibroblast-like phenotype.^8^ Molecular dissection identified SMAD signalling as the driving force for this differentiation; SMAD regulates the repression of epithelial markers and activation of mesenchymal markers.^9^ In addition to TGF-β, Snail, Slug, E12/E47, ZEB-1 and SIP-1 regulate EMT in different cancer cell lines.^10,11^ We have previously shown that transcription factors Twist and FOXC2 also play critical roles in EMT and metastasis of breast tumours.^12,13^ SNAI1/2, PRRX1, EZH2 and miRNAs such as the miR-200 family, miR-34 and miR-141 are important during the EMT as well.^2,14^ These trans-regulatory factors drive EMT through feedback and feedforward mechanisms. Tumour cells that have undergone EMT exhibit stem-cell-like properties, including the ability to self-renew, tumour-initiation properties and resistance to chemotherapy and radiotherapy. Ovarian cancer cells express EMT-related genes as well as stem-cell markers such as CD44, ALDH1A1, Nanog, SOX2, Notch1/4, Oct4 and Lin28, and these markers can be used to identify metastatic and therapy-resistant tumours.^15^

Our understanding of EMT has increased substantially over the last decade, but the process of MET in the later stages of metastasis is less well characterised.^16^ The overexpression of the fibroblast growth factor receptor FGFR2IIIC in a bladder cancer cell line resulted in MET,^17^ and in the breast cancer cell line (MDA-MB-231), overexpression of ERp29 lead to MET transition with loss of mesenchymal markers and induced expression of E-cadherin.^18^ Downregulation of MMP-7 and laminin-5γ2 and resurgent E-cadherin expression, indicative of MET, was observed in lung parenchyma metastatic cells.^19^ Moreover, multiple lines of evidence suggest that the cellular machinery required for EMT induction is closely related to that necessary for MET including TGF-β superfamily members, bone morphogenetic proteins (BMP2, BMP7), AKT, WIF1 and FZD4.^20–24^ miRNAs also contribute to MET. For example, in oesophageal squamous cell carcinoma, miR-150 induces MET-like changes by inducing degradation of *Zeb-1* mRNA, inhibiting tumorigenicity and tumour growth in a mouse xenograft model.^25^ Research on the EMT and MET has also uncovered numerous novel signalling pathways, including TGF-β, Wnt, Notch, Hedgehog and PI3K pathways, that facilitate EMT in tumour cells.^16,22^

Given the complexity of the EMT and MET transition process, understanding the true driving factors and nature of epithelial and mesenchymal states remains a major challenge in the research community. Although our understanding of the epithelial and mesenchymal state is based on molecular and phenotypic data available in the public domain, the information is scattered in the literature. While existing EMT databases such as dbEMT and EMT-Regulome provide a resource for the understanding EMT, their usage across cancers, and cross interrogation with other omics platforms is limited.^26,27^ In light of this, we gathered available signatures associated with EMT and MET transition from the literature. For example, we reported the derivation of an EMT core signature by overexpressing EMT-inducing TFs in breast tumour cell lines, which correlated strongly with poor survival of breast cancer patients.^28^ In prostate cancer, a signature of 49 genes was identified in CTCs, which were associated with metastatic castration-resistant prostate cancer.^29^ In 113 colon cancer patients, 33 molecular determinants were associated with an EMT signature.^30^ Meta-analysis of 54 lung cancer (NSCLC) cell lines identified a 76 gene EMT signature that classified cell lines into distinct epithelial- and mesenchymal-like groups independent of array platform.^31^ Soundararajan et al., reported a cell plasticity associated novel prognostic gene expression signature derived from mouse embryonic day 6.5.^32^ Metabolic analysis has further identified a unique panel of metabolites associated with EMT in breast cancer cell lines.^33^ A pan-cancer analysis identified a 77 gene signature from 11 cancer types (TCGA, *n* = 1104) associated with EMT and its clinical relevance.^34^ Whereas, interrogating the EMT spectrum in ovarian cancer revealed a 33 gene EMT signature associated with an intermediate subgroup with worse progression-free survival.^35^ Single-cell analysis from 18 head and neck squamous cell carcinoma (HNSCC) patients identified a gene signature for partial EMT (p-EMT) used to differentiate samples with an adverse phenotype.^36^ Although the expression of markers or signatures was widely used to determine the EMT and MET state in a given cancer type, our understanding is still limited.

To serve the need for a comprehensive EMT multiomics platform for the research community in primary and metastatic research, we developed the EMTome portal. The EMTome includes EMT and MET related genes and signatures across cancer types, possible interacting partners, including miRNAs, transcription factors and kinases. We used publicly available data from Cancer Cell Line Encyclopedia (CCLE),^37^ the Cancer genome atlas (TCGA) cancer cohorts consisting of more than 10,000 patients with primary or metastasis cancer, and metastatic cohort (MET500)^38^ to explore transcriptomics, epigenomics, mutation, immunome and clinical relevance of identified genes/signatures. Apart from genomic features, EMTome helps to interrogate genetic dependency based on CRISPR/CAS9 dataset^39^ and drug sensitivity from the genomics of drug sensitivity (GDSC).^40^ EMTome is the first of its kind, to our knowledge, providing a unique platform to the research community to query, access, and analyse EMT/MET signatures across cancer cohorts.

## Methods

### EMT/MET core signatures

We identified and collected EMT/MET gene signatures from the literature. We used the terms “(Epithelial OR Mesenchymal) AND (EMT OR MET) AND signature AND Cancer” to search the PubMed database for all relevant articles. We retrieved 445 publications (searched Jan 24, 2020) with the EMT-signature term from more than 27,000 publications on EMT (Fig. S1a, Dataset 3), and identified EMT signatures with a minimum of three genes as the cut-off. Signature information and genes associated with the signature were retrieved from the articles by one of the authors. In total, 810 protein-coding and 122 noncoding genes have been associated with EMT/MET. The database is gene/protein-centric with gene information retrieved from NCBI with the Entrez Gene symbol as the key identifier. Furthermore, EMT-related genes not found in the signature, but identified in the literature, were collected using GLAD4U^41^ with the search query “Epithelial-mesenchymal transition, EMT, cancer”. In total, 314 genes were selected from 4,499 publications using a hypergeometric test to rank genes.

### Cell line RNA expression dataset

We retrieved the RNA expression dataset for cell lines from the Cancer Cell Line Encyclopedia (CCLE).^37^ The dataset consists of RNA-seq gene expression data from 1019 cell lines at RNA-Seq by Expectation-Maximization (RSEM) transcripts per million level. The cell lines were developed from different cancers: acute myeloid leukaemia, B-cell lymphoma, breast, cervix, colorectal, endometrium, oesophagus, glioma, kidney, liver, non-small-cell lung, small cell lung, Burkitt’s lymphoma, medulloblastoma, melanoma, mesothelioma, multiple myeloma, neuroblastoma, osteosarcoma, ovary, pancreas, prostate, stomach, T-cell acute lymphoblastic leukaemia, T-cell lymphoma, thyroid, upper aerodigestive and urinary tract (https://portals.broadinstitute.org/ccle).

### TCGA patient multiomics dataset

We downloaded patient genomic, epigenomic, and transcriptomic data for 32 TCGA cancer types from the Firehose of the Broad Institute (http://gdac.broadinstitute.org/, January 2016 version). The datasets include mutation, copy number alteration (CNA), methylation, mRNA expression, miRNA expression and reverse-phase protein array (RPPA) data, which are normalised. Clinical data with overall survival time, vital status and tumour stage were also extracted. Cancer cohorts included were: adrenocortical carcinoma, bladder urothelial carcinoma, breast invasive carcinoma, cervical and endocervical cancers, cholangiocarcinoma, colon cancer, lymphoid neoplasm diffuse large B-cell lymphoma, oesophageal carcinoma, glioblastoma multiforme, head and neck squamous cell carcinoma, kidney chromophobe, kidney renal clear cell carcinoma, kidney renal papillary cell carcinoma, acute myeloid leukaemia, brain lower-grade glioma, liver hepatocellular carcinoma, lung adenocarcinoma, lung squamous cell carcinoma, mesothelioma, ovarian serous cystadenocarcinoma, pancreatic adenocarcinoma, pheochromocytoma and paraganglioma, prostate adenocarcinoma, rectal adenocarcinoma, sarcoma, skin cutaneous melanoma, stomach adenocarcinoma, testicular germ cell tumours, thyroid carcinoma, thymoma, uterine corpus endometrial carcinoma, uterine carcinosarcoma, uveal melanoma. The pan-cancer batch effect normalised RNA expression (log2(normalised value+1)), immune phenotype annotation,^42^ stemness score^43^ and curated survival endpoints^44^ were retrieved from XENA (https://xenabrowser.net/).

### Metastatic cancer patient data

Data on 500 cancer patients with metastatic disease from 30 primary sites and biopsied from 22 organs was obtained from MET500 dataset using XENA.^38^ The RNA expression for 868 cases was available in fragments per kilobase of exon model per million reads mapped format.

### Signature-derived normalised enrichment score (NES)

The cancer hallmark enrichment score for each patient in a cancer cohort was inferred by the ssGSEA method implemented in GSVA using a hallmark signature from MSigDB (http://software.broadinstitute.org/gsea/msigdb, v7.0).^45^ Immune abundance was inferred for each patient using geneset markers previously published.^46^ An EMT-signature score was calculated using EMT-signature genes obtained from relevant articles. We used the geneset enrichment analysis (GSEA) method to calculate the EMT-signature score (EMT NES), for this we used RNA-seq data (RSEM, gene-level log2-transformed) from the relevant cancer type (TCGA). Over-representation of cancer hallmark gene enrichment analysis was performed using WebGestalt,^47^ and gene ontology enrichment was performed using Metascape.^48^ The KEGG pathway analysis was performed using clusterProfiler v3.11.^49^

### CRISPR/Cas9 loss-of-function and drug screening data

The gene dependency data collected by Project Achilles was obtained from the Cancer Dependency Map to interrogate EMT-related gene-based cancer vulnerabilities across cancer types. The data consist of an in vitro study of genetic dependencies in cancer cell lines using CRISPR/Cas9 loss-of-function screens (https://depmap.org).^39^ Further, the cancer cell line sensitivity to various drugs was obtained from The Genomics of Drug Sensitivity of Cancer (GDSC) database (Release 8.2, 2020).^40^ The data consists of drug sensitivity data of 988 cancer cell lines and 518 compounds from GDSC1 and GDSC2 datasets. The IC_50_ values in cancer cell lines categorised into primary or metastatic phenotypes were extracted.

### Survival analysis

Normalised gene expression was categorised into low and high expression based on the average expression. Curated survival endpoints used for analysis each gene expression profile were overall survival (OS), disease-specific survival (DSS), disease-free interval (DFI) and progression-free interval (PFI).^44^ The hazard ratios across cancer types were shown in dumb-bell plot. To compare survival response across EMT signatures and cancer types, we used normalised enrichment score of the EMT signature and categorised into low and high expression based on the median. For overall survival we focused on 33 cancer types of TCGA cohorts. While for progression-free interval (PFI) survival we focused on 32 cancer types due to unavailability of Acute Myeloid Leukaemia (LAML) PFI. Univariate Cox proportional hazards models were fitted to calculate the hazard ratios using *coxph* function in the Survival package (version 2.44).

### Statistical analysis

The statistical analyses were performed using the computing environment R (3.5.2). The correlations between hallmark or immune cell abundance were determined in pan-cancer using Spearman’s correlation function implemented in R. The correlation coefficient and *p* values were retrieved. In all cases, *p* values less than 0.05 were considered statistically significant.

### Implementation

The EMTome web interface was developed using HyperText Markup Language (HTML) and the Hypertext Preprocessor (PHP). Users can access the webpage through the web link www.emtome.org. The signature dataset and related gene sets are stored as tables that can be accessed through the web interface. The statistical analysis was precalculated to avoid delays in data processing. However, for visualisation functionality at the cohort level, we provide on-the-fly analysis using computing environment R (3.5.2) on the server side.

## Results

### EMTome modules

EMTome is comprised of four modules: EMT core signature, EMT-related gene retrieval, EMT-related gene interactome, and EMT-signature score (Fig. 1b). The EMT core signature module allows users to explore the known signatures in a given cancer type and associated genes or gene products. The EMT-related gene retrieval module allows searches of genomic, epigenomic, transcriptomic, proteomic and survival information for a gene of interest. The EMT-related gene interactome module can be used to identify significant associations between RNA expression levels and the transcriptomic landscape, copy number events, DNA methylation, miRNA and proteins (RPPA). The fourth module, EMT-signature score, can be used to determine correlations between the normalised EMT score at the patient level and respective RNA expression, to make survival comparisons among signatures across cancer types, and to evaluate the genetic dependency of EMT-related genes and associated drug sensitivity information (Fig. 1c).

### EMT core signature database

We have reported that EMT, which is activated during cancer progression, not only facilitates tumour cell colonisation of other organs but also induces resistance to traditional chemotherapy.^50^ EMT is a complex process activated by multiple molecular switches. We have previously shown that TWIST1 and FOXC2 play key roles during the EMT process; other such factors were discovered recently.^12,13,51^ Given the complexity of the EMT process, signatures defined in a particular cancer rarely overlap with signatures obtained from other cancer types or organs. Furthermore, data available in the public domain are scattered in the literature and not easily accessible (Fig. 1a, b). In light of this, we performed data mining to collect EMT/MET signatures from the literature. We defined an EMT signature as three or more genes in combination or a unique pattern of expression responsible for either EMT or MET. We retrieved 83 gene-level EMT signatures from the literature (Dataset 1). The median number of genes in these signatures was 33 with a range from 3 to 593. These signatures and gene markers are available in tabular view as accessible resources.

### EMT-related gene retrieval

**Fig. 2:**
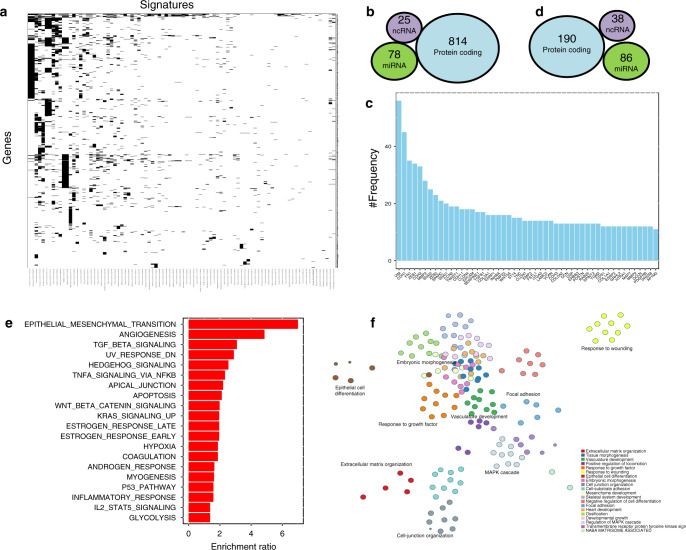
EMT core signatures and associated markers. **a** Overlap among markers associated with each signature. **b** Numbers of protein-coding, miRNA, and other noncoding RNA genes found in more than three or more signatures. **c** The enrichment ratios of genes present at high frequency across signatures. **d** Numbers of protein-coding, miRNA, and other noncoding RNA genes identified with significant frequency in the literature (based on PubMed search February 2020). **e** Cancer hallmark enrichment analysis shows EMT, angiogenesis, TGF-β signalling pathway and apical junction related terms enriched for EMT-related genes (*p* < 0.05). **f** Over-representation of gene ontology terms shows enrichment for extracellular matrix organisation, tissue morphogenesis, vasculature development, positive regulation of locomotion, response to growth factor, epithelial cell differentiation and embryonic morphogenesis.

From the collected EMT-signature dataset, we retrieved gene markers associated with each signature, resulting in 3600 protein-coding and noncoding genes. The genes were annotated with the accepted NCBI Entrez gene symbol and the extent of overlap among the signatures was evaluated (Fig. 2a). Of the 3600 protein-coding and noncoding genes in the signature, 814 were found in three or more signature gene sets (Fig. 2b). Several miRNAs and other noncoding RNAs were also found in three or more of the gene signatures (Fig. 2b). A number of genes from each of these categories were detected at high frequency (Fig. 2c).

We next used the GLAD4U gene retrieval and prioritisation and identified 314 genes mentioned with significant frequency in the literature as being correlated with EMT (see Methods; Fig. 2d). We combined the signature-derived and literature-identified EMT-related markers, which resulted in EMT-related 936 protein-coding genes, 61 noncoding RNAs, and 156 miRNAs (Dataset 2). Cancer hallmark enrichment analysis showed that these genes were enriched in terms related to EMT, angiogenesis, TGF-β signalling pathway and apical junctions (*p* < 0.05, Fig. 2e). Further, over-representation analysis for gene ontology terms showed that these genes were enriched for terms related to extracellular matrix organisation, tissue morphogenesis, vasculature development, positive regulation of locomotion, response to growth factors, epithelial cell differentiation and embryonic morphogenesis (Fig. 2f), supporting roles in EMT or MET.

For each gene, information was retrieved from a public cohort, like TCGA, and linked through EMTome. Information includes mutations, variants, copy number, mRNA expression, protein expression, methylation and overall patient survival for 32 cancer types. Further, EMT-related gene RNA expression and correlations with hallmark enrichment are given tabulated. The correlations can be visualised as scatter plots for each hallmark in a cancer type with stage level information in a grid plot.

EMTome visualisation will help users perform experimental analyses or validate their hypothesis. For example, we used EMTome to perform sample-level immune abundance enrichment for TCGA cancer type and correlated with *SNAI1* expression. Levels of *SNAI1*, which encodes a transcriptional repressor associated with EMT, are correlated positively with the hallmark of EMT in kidney chromophobe carcinoma (Spearman *ρ* = 0.63, *p* < 0.001; Fig. S2a). The association increases with the stage in kidney cancer. Metastasis was shown to be associated with poor immune infiltration,^52^ and analysis using EMTome revealed that *SNAI1* expression positively correlates with myeloid-derived suppressor cell frequency (Spearman *ρ* = 0.69, *p* < 0.001, Fig. S2b) but poorly correlates with activated CD4 T cells (*ρ* = −0.39, *p* = 0.001; Fig. S2c Dataset 3) and CD8^+^ T cell (*ρ* = −0.25, *p* < 0.05; Fig. S2d Dataset 3) frequencies. We also discovered associations between *SNAI1* expression and cancer hallmarks (Fig. S2e) and immune cell markers (Fig. S2f) across cancer types. Similar results were observed where SNAI1 was shown to generate immunosuppressive cells to induce metastasis, suggesting possible therapeutic opportunities that may disrupt immunosuppressive cells.^53–55^

### EMT-related gene interactome

**Fig. 3:**
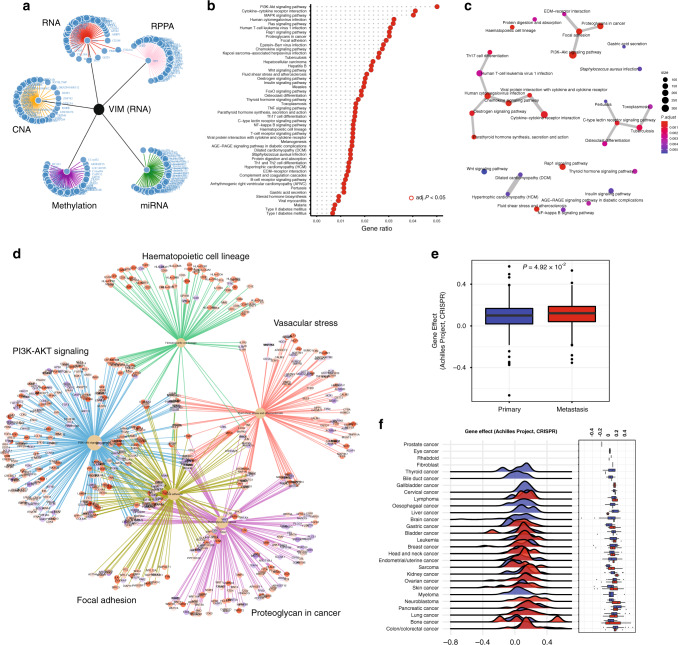
VIM interactome. **a** The genes, miRNAs, copy number alterations, methylated genes and proteins associated with VIM in bladder cancer (the top 50 from each category are shown). **b** The KEGG pathway enrichment terms for genes significantly correlated with VIM and **c** their overlap. **d** Enrichment terms most highly linked with VIM and associated genes. **e** Differential VIM gene dependency between primary and metastatic cell lines using CRISPR/Cas9 knockout and across cancer types (**f**).

Although association analysis only identifies the strongly correlated features, it can also facilitate the identification of possible causative features.^56^ Using EMTome, the user can explore the association of EMT-related gene expression with expression of related genes, alteration of copy number events, methylation or miRNA expression or protein levels (RPPA) (Fig. S3a). For example, associations of VIM with genes, gene products, miRNAs, copy number alterations, methylation and protein levels within bladder cancer were explored (Fig. S3b, Dataset 3). The network of these associations can be readily visualised (top 50 in each platform, Fig. 3a). The KEGG pathway enrichment for the TWIST1-correlated genes suggests enrichment of the PI3K-AKT signalling pathway, proteoglycans in cancer, RAS signalling pathway and focal adhesion (Fig. 3b–d). All the associations based EMT-related gene interactome results were calculated using the Spearman correlation and filtered with a false discovery rate (FDR) adjusted *p* value at 0.01 (Benjamini–Hochberg correction). Users can easily access the significant interactome results for each TCGA cancer cohort through the webpage.

Furthermore, the user can explore the gene dependency of EMT-related genes using CRISPR/Cas9 loss-of-function data or drug sensitivity data. For example, VIM is highly expressed in metastatic tumours including bladder cancers with high tumour grade and stages.^57^ Linking to CRISPR/Cas9-based *VIM* knockout data revealed that vimentin dependency across cell lines with metastatic properties is higher than in cell lines without metastatic properties (*p* = 4.92 × 10^−2^, Fig. 3e). Furthermore, the loss-of-function of VIM across primary and metastatic cell lines can be explored to interrogate gene effect across cancer types (Fig. 3f).

### EMT-signature score

An EMT-signature score is calculated using the signature geneset (see Method section). In the EMT score panel, users can select a gene of interest and an EMT signature and submit a query to evaluate the relationships among them. The query processes the gene expression at the RNA level and matched EMT-signature score obtained at the patient level within various cancer cohorts. In the EMT-signature score module, the scoring does not consider the association of a gene with the epithelial or mesenchymal phenotype; thus, the association needs to be interpreted based on the signature phenotype. To address this, we selected top epithelial and mesenchymal genes (top 50 based on frequency in combined signatures) and calculated normalised enrichment scores similar to EMT signatures. Users can explore the relationship between EMT-related genes and signature along with the top 50 epithelial and mesenchymal signatures to conclude their findings. Further, to demonstrates the utility of the EMT signature in a cancer cohort, we performed survival analysis using clinical features (progression-free interval and overall survival) and EMT-signature scores obtained at the patient level. Users can explore the prognostic utility of the EMT-signature across cancer types using cox regression model and can be visualised as survival forest plot (Fig. S4, Dataset 3).

### Case study

#### Pathway representation of EMT signatures

**Fig. 4:**
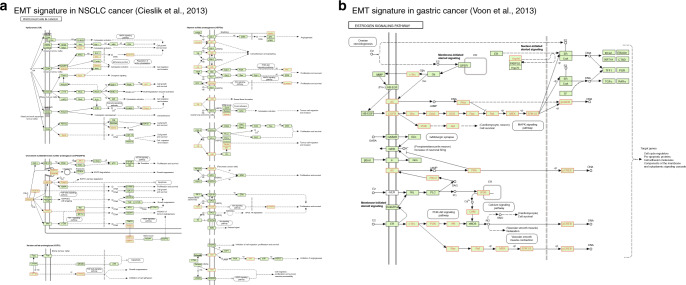
Pathway enrichment of EMT signatures. **a** Cieslik et al. EMT signature consisting of 141 gene enriched in proteoglycans in cancer pathway (*p* < 0.001). **b** Voon et al. EMT signature consisting of 93 genes from gastric cancer enriched in oestrogen signalling pathway (*p* < 0.001). The highlighted genes were observed in signatures.

EMT signatures identified by authors represent a resource of possible biomarkers in pan-cancer analysis and can provide their functional relevance in tumours. Thus, we performed geneset enrichment analyses for KEGG pathways, which have been widely used to explore the role of pathways from gene list^49,58^ and provided it on the webportal under each EMT-signature profile. The pathway enrichment was calculated using a hypergeometric test based on whether input geneset had a different frequency of annotation pairs unlikely by chance. For example, Cieslik et al. have identified 141 gene signatures associated with non-small-cell lung cancer.^59^ The signature genes were enriched with proteoglycans in cancer (*p* < 0.001), Hippo signalling pathway (*p* < 0.001), focal adhesion (*p* < 0.001) and leukocyte trans-endothelial migration (*p* < 0.001). Among proteoglycans control morphogenesis, vascularisation and cancer metastasis and is markedly expressed in the tumour microenvironment during tumour development.^60^ The proteoglycans in cancer is shown in Fig. 4a. The enriched proteoglycans genes in different cancer types, including lung cancer suggest a therapeutic opportunity in targeting the EMT process.^61^ On the other hand, Voon et al. have derived an EMT signature consisting of 93 genes from a microarray dataset of gastric cancer showing enrichment for endocrine resistance (*p* < 0.001), oestrogen signalling (*p* < 0.001), GnRH signalling (*p* < 0.001) and ErbB signalling (*p* < 0.001) pathways apart from EGFR/RAS pathway.^62^ The enrichment of the oestrogen signalling pathway in gastric cancer suggests a possible endocrine therapy approach, which was shown to impact cell migration negatively and enhance chemosensitivity (Fig. 4b).^63,64^ Although we observed an enriched endocrine resistance pathway, it was majorly associated with an altered ER pathway. Overall, the enrichment and visual representation of EMT-signature genes provides a useful tool to biologists.

#### FGFR1 loss-of-function in primary and metastatic cell lines

**Fig. 5:**
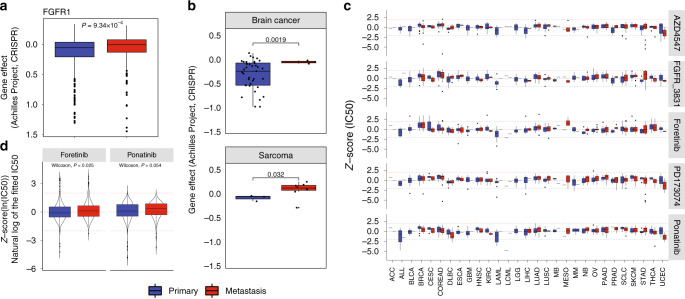
FGFR1 gene dependency in primary and metastatic cell lines. **a** FGFR1 differential dependency in primary and metastatic cell lines (Wilcoxon test, *p* = 9.34 × 10^−4^). **b** We observed higher FGFR1 dependency in metastatic cell lines compared to primary including Brain cancer (*t*-test, *p* = 1.9 × 10^−3^) and sarcoma (*t*-test, *p* = 0.032). **c** The relative comparison of IC50 between different cancer cell lines treated with FGFR1 inhibitor (AZD4547, FGFR3861, Foretinib, PD173074, Ponatinib) with significant difference in primary and metastatic cell lines. **d** Foretinib and Ponatinib drug molecules effective in most of metastatic cancer cell lines compared to primary tumour cell lines.

To explore the impact of EMT-related genes in primary and metastatic cell lines, we interrogated gene dependency using CRISPR/Cas9 loss-of-function dataset (Depmap portal^65^). Previous reports have demonstrated increased expression of fibroblast growth factor receptor 1 (FGFR1) during EMT and FGFR1 plays a key role in metastatic tumour growth and is suggested as a mechanism of resistance in targeted molecular therapies.^66–68^ Given these previous findings, we observed FGFR1 differential dependency in primary and metastatic cell lines (Fig. 5a, *p* = 9.34 × 10^−4^). We observed higher FGFR1 dependency in metastatic cell lines compared to primary, including brain cancer (*p* = 1.9 × 10^−3^) and sarcoma (*p* = 0.032) as shown in Fig. 5b. To identify therapeutic opportunity in different cancer types, we interrogated the FGFR1 drug screen in various cancer cell lines. The relative comparison of IC50s between different cancer cell lines treated with FGFR1 inhibitor (AZD4547, FGFR3861, Foretinib, PD173074, Ponatinib) with a significant difference in primary and metastatic cell lines are shown (Fig. 5c). Overall, we found Foretinib and Ponatinib to be effective in most of metastatic cancer cell lines compared to primary tumour cell lines, suggesting therapies to target FGFR1 in metastatic cancer (Fig. 5d).

#### Prognostic utility of EMT signature across cancer types

**Fig. 6:**
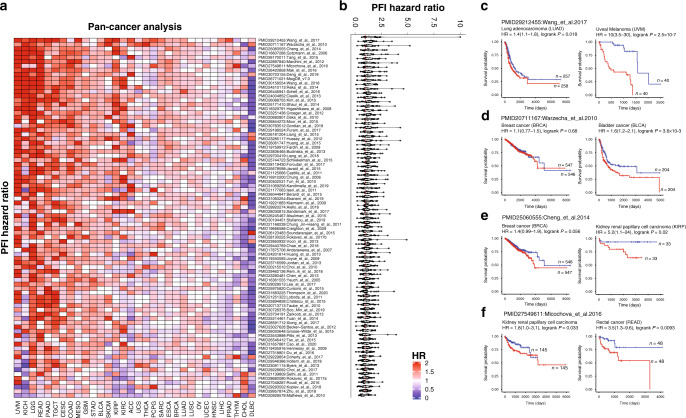
Prognostic utility of EMT-signatures. **a** Progression-free survival analysis for EMT signature with hazard ratio shown in heatmap across 32 cancer types. **b** Boxplot representing the hazard ratio for 32 cancer types where each dot characterises a cancer type. Wang et al. 2017 EMT signature in lung cancer based on network analysis found to be associated with poor survival in multiple cancers. **c** Wang et al. signature-based progression-free survival in lung adenocarcinoma (HR = 1.4, logrank *p* = 0.018) and uveal melanoma (HR = 10, logrank *p* < 0.001). **d** Warzecha et al signature-based progression-free survival in breast cancer (HR = 1.1, logrank *p* = 0.68) and bladder cancer cohort (HR = 1.6, logrank *p* = 3.6 × 10^−3^). **e** Cheng et al. signature-based progression-free survival in breast cancer (HR = 1.4, logrank *p* = 0.056) and kidney renal papillary cell carcinoma cohort (HR = 5.2, logrank *p* = 0.02). **f** Mlcochova et al. 2016 signature-based progression-free survival in kidney renal papillary cell carcinoma (HR = 1.8, logrank *p* = 0.033) and rectal cancer cohort (HR = 3.5, logrank *p* = 9.3 × 10^−3^).

Although EMT signature associated genes represent the markers associated with the EMT process and led to metastasis, we observed heterogeneity in the EMT-signature genes across publications and cancer type. To correlate the signatures with the patient’s survival, we performed a progression-free survival analysis for each signature consisting of EMT-related biomarkers across 32 cancer types (Fig. 6). The hazard ratio for each EMT signature across cancer types shown in heatmap suggests differential prognosis across cancer types (Fig. 6a). Many of the signatures were found to be significantly associated with poor prognosis in uveal melanoma (UVM), brain cancer (GBM, LGG), colorectal adenocarcinoma (COAD, READ), pancreatic adenocarcinoma (PAAD), Testicular cancer (TGCT) and cervical cancer (CESC) but not in lymphoid cancer (DLBC). Among EMT signatures, we observed EMT signature (Wang et el. 2017) in lung cancer based on network analysis to be associated with poor progression-free survival in multiple cancers followed by ESRP-regulated splicing EMT signature (Warzecha et al. 2010), EM plasticity signature (Cheng et al. 2014) and EMT signature in Hepatocellular carcinoma (Gotzmann et al. 2006) (Fig. 6b). Wang et al. signature was associated with poor survival in lung adenocarcinoma (Fig. 6c, HR = 1.4, logrank *p* = 1.8 × 10^−2^) and significantly higher in uveal melanoma (Fig. 6c, HR = 10, logrank *p* < 0.001). Warzecha et al. signature was associated with poor survival in breast cancer but not significant (Fig. 6d, HR = 1.1, logrank *p* = 0.68) but significant in bladder cancer cohort (Fig. 6d, HR = 1.6, logrank *p* = 3.6 × 10^−3^). Cheng et al. signature was associated with poor survival in breast cancer (Fig. 6e, HR = 1.4, logrank *p* = 5.6 × 10^−2^) but significant in kidney renal papillary cell carcinoma cohort (Fig. 6e, HR = 5.2, logrank *p* = 2 × 10^−2^). Mlcochova et al. (2016) signature was significantly associated with poor survival in Kidney renal papillary cell carcinoma (Fig. 6f, HR = 1.8, logrank *p* = 3.3 × 10^−2^) as well as rectal cancer cohort (Fig. 6f, HR = 3.5, logrank *p* = 9.3 × 10^−3^). Overall, the pan-cancer survival analysis provides a unique opportunity to identify the consistent role of EMT signatures.

#### User interface and information retrieval

EMTome has a simple and intuitive user interface for data access and exploration of the EMT signatures and EMT-related genes across cancer types (Fig. S5a–c). The interface enables the user to evaluate transcriptomic associations with omics platforms to retrieve and visualise significant associations (Fig. S5d). Genes are hyperlinked to enable retrieval of detailed information through NCBI GeneCards. EMT scores can be calculated based on EMT signatures across the pan-cancer cohort (Fig. S5e), and loss-of-function and drug sensitivity data can be explored (Fig. S5f). Download options allow users to download analysis results in PNG or Excel format or in tabular form (Fig. S5g). Relevant gene sets can also be downloaded as text or in GMT file format from the same interface.

## Discussion

The EMTome resource was designed to enhance our understanding of EMT signatures and integrate current knowledge into a single platform to provide genomic, transcriptomic, epigenomic, mutation, immune, proteomic and clinical information for EMT-related genes. EMTome goes beyond currently available resources, dbEMT and EMT-regulome^26,27^ with data of many cancer types and integration with different omics platforms. EMTome can be used for: (i) exploration of predicted EMT signatures in cell lines and model organisms, (ii) retrieval of detailed information on EMT-related gene obtained through various high-throughput ‘omics analyses across cancer cohorts, (iii) determination of the EMT-related gene interactome based on statistical significance from transcriptome, epigenome and proteomic analyses, (iv) comparison of EMT signatures in cancer cohorts and their associations with EMT-related genes, and visualisation and download of results.

Currently, EMTome incorporates information from the CCLE dataset consisting of 1094 cell lines, a metastatic dataset with 500 patients, and the TCGA database with information on more than 11,000 patients. Also included is human proteome atlas immunohistochemistry data on EMT-related gene expression in independent cancer cohorts. EMTome further provides functional information for each signature in a TCGA cancer cohort and related omics analysis data that allow users to explore possible roles of genes of interest in cancer. Notably, the mutation plot is useful for detecting genomic aberrations and their post-translational modifications. The bar graph for copy number variation allows the user to investigate alterations such as deletions or amplifications of the gene of interest in a given cancer cohort. The RNA expression bar graph can be used to plot the abundance of the gene of interest across all cancer cohorts. The distribution of RNA abundance can also be explored at the cell line level and the protein expression level. Proteomics data can be accessed using RPPA data. In addition to gene expression in primary tumours, EMTome includes gene expression profiles in metastatic cell lines and patient samples. The cancer hallmarks, immune enrichment and stemness association with EMT-related genes can be retrieved as scatter plots, and immune cluster correlations with EMT-related genes and subtype-specific enrichment as boxplots. Data from the TCGA cohort, including overall survival, disease-specific survival, disease-free interval and progression-free interval, can be evaluated in terms of the gene of interest. In addition to gene-specific information, an EMT-related gene interactome, based on the association of RNA expression with other transcriptomic features, copy number events, DNA methylation, miRNAs and proteins (RPPA) can be evaluated using EMTome. To understand how the loss of function or drug inhibition influences cancer cell lines, we included data on genetic dependency and drug sensitivity for EMT-related genes in primary and metastatic cancer cell lines. Further, we provided three case studies to help users explore the webportal for their research.

In summary, EMTome provides a comprehensive resource for identifying metastasis-related features, exploring EMT-related markers, and for analysis of the relevance of EMT signatures for diagnosis or prevention of cancer metastasis. The user interface and database structure would allow the database to be extended to additional cancer types. Currently, EMTome lacks high-dimensional proteomics and Chip-seq data, but this type of data could be included with availability from on-going studies in updated versions. Although the current version of EMTome includes only cell line data from CCLE and patient data from TCGA and the MET500 and HPA datasets, it can be easily extended to support other cohort-based datasets.

## Supplementary information

Dataset 1

Dataset 2

Dataset 3

Supplemental Material

## Data Availability

The data supporting the results are in the public domain and mentioned in methods.
