# Supplementary material for: EMTome: a resource for pan-cancer analysis of epithelial-mesenchymal transition genes and signatures
Source: Br J Cancer. 2020 Dec 10;124(1):259–69. doi: 10.1038/s41416-020-01178-9 (PMC7782839; doi:10.1038/s41416-020-01178-9)
Supplement: Supplementary file 4 — Supplemental Material [file 41416_2020_1178_MOESM4_ESM.doc]

**SUPPLEMENTARY DATA**

**Supplementary Figure S1.** **EMT signatures and EMT-related publications.** (a) As of Jan 24, 2020, 502 publications had an EMT signature term as a keyword. (b) Approximately 27,000 publications contained the word EMT.

**Supplementary Figure S2.** **Association of *SNAI1* expression with EMT, cancer hallmarks, and immune cell enrichment scores.** *SNAI1* expression positively correlated with a) hallmarks of EMT (Spearman’s 𝜌=0.63, p<0.001) and (b) immune myeloid-derived suppressor cell infiltration (Spearman’s 𝜌=0.69, p<0.001) in kidney chromophobe carcinoma in a stage-dependent. (c) Association between *SNAI1* expression and cancer hallmarks. (d) Association between *SNAI1* expression and immune cell frequencies across cancer types. (e) SNAI1 gene expression association with CD4 T and (f) CD8 T cells in TCGA Kidney chromophobe carcinoma (KICH).

**Supplementary Figure S3.** (a) EMT-related multiple ‘omics platforms based interactome. (b) VIM-associated genes or gene products, miRNAs, copy number alterations, methylated genes, or proteins with enrichment in bladder cancer (n=408) (Benjamini-Hochberg correction, FDR < 0.05).

**Supplementary Figure S4.** **EMT-signature survival assessment in breast cancer.** Comparative Progression free and overall survival analysis of EMT-signatures (n=84) based on cumulative EMT-signature gene set at the patient level in a breast cancer cohort (n=1093) shown as a survival forest plot. Also shown are Cox-regression coefficient, model-based hazard ratio, log rank *p* value, and significance of the signature.

**Supplementary Figure S5. A simple and intuitive user interface to access the EMTome modules.** (a) The home page of the EMTome. (b) Interface for exploration of EMT signatures across human cancers. (c) Interface for exploration of EMT-related genes and their genomic, transcriptomic, proteomic, immune, and survival profiles in 32 cancer types. (d) Interface to provides ‘omics data. (e) Interface for calculations of EMT-signature scores based on gene set enrichment analysis across pancancer cohort. (f) Interface for exploration of CRISPR/Cas9 knockdown and drug sensitivity data. (g) Interface for download options to access the signature in a simple tabular format.
